# The use of the global activity limitation Indicator and healthy life years by member states and the European Commission

**DOI:** 10.1186/s13690-018-0279-z

**Published:** 2018-06-28

**Authors:** Petronille Bogaert, Herman Van Oyen, Isabelle Beluche, Emmanuelle Cambois, Jean-Marie Robine

**Affiliations:** 1 Department of Public Health and Surveillance, Epidemiology and public health, Sciensano, Rue Juliette Wytsmanstraat 14, 1050 Brussels, Belgium; 2 Tilburg University, Tilburg, The Netherlands; 3 Ghent University, Ghent, Belgium; 4 French Institute for Demographic Studies (INED), Paris, France; 5 French Institute of Health and Medical Research (INSERM), Paris, France; 6 École Pratique des Hautes Études, Paris, France

**Keywords:** Global activity limitation Indicator, Disability-free life expectancy, Healthy life years, Disability, Policy

## Abstract

**Background:**

In 2005, the European Union (EU) started to use a disability-free life expectancy, known as Healthy Life Years (HLY), to monitor progress in the strategic European policies such as the 2000 Lisbon strategy. HLY are based on the underlying measure: the Global Activity Limitation Indicator (GALI). Twelve years after its implementation, this study aims to assess its current use in EU Member States and the European Commission.

**Methods:**

In March 2017, a questionnaire was sent to 28 Member states and the European Commission. The questionnaire inquired how the GALI and HLY are used to set policy targets, in which surveys the GALI has been introduced since 2005, how the GALI and HLY are presented, and what the capacity in each country is to investigate the GALI and HLY.

**Results:**

The survey was answered by 22 Member States and by the Commission. HLY are often used to set targets and develop strategies in health such as national health plans. Analysis of HLY has even led to policy change. In some countries, HLY have become the main indicator for health, gaining more importance than life expectancy. More recently, the GALI and HLY have also been used for policy targets outside the health sector such as in the area of pension and retirement age or in the context of sustainable development. Regarding surveys, the GALI is mostly obtained from the EU-SILC, SHARE and EHIS, but is also increasingly introduced in national surveys. National health reporting systems usually present HLY on their national statistics websites. Most countries have up to three specialists working on the GALI and HLY, which has been consistent through time. Others have increased their capacity over various institutions.

**Conclusion:**

HLY is an indicator that is systematically used to monitor health developments in most EU countries. The SHARE, EU-SILC and EHIS are commonly used to assess HLY through the GALI. The results are then described in reports and presented on national statistics websites and used in different policy settings. Expertise to analyse the GALI and HLY is available in most countries.

**Electronic supplementary material:**

The online version of this article (10.1186/s13690-018-0279-z) contains supplementary material, which is available to authorized users.

## Background

Health expectancies combine information on mortality and morbidity, where the quality of remaining life is as important as the quantity [1]. Increasingly, health expectancies are being used as summary measures of population health, serving as an essential indicator of the health of the ageing populations [2]. Healthy Life Years (HLY), a disability-free life expectancy, measures the expected years lived without participation restriction. In Europe, it has been monitored since 2005 and was selected to help measure progress in strategic European policies such as the 2000 Lisbon strategy and the European strategy on Active and Healthy Ageing [3]. HLY are based on the underlying measure: the Global Activity Limitation Indicator (GALI) which is provided through surveys. The question is: “*for at least the past six months, to what extent have you been limited because of a health problem in activities people usually do? Would you say you have been: severely limited? limited but not severely? not limited at all*?”. The development of the GALI occurred in the framework of the creation of a coherent set of indicators to monitor health across Europe [4]. The GALI is one of the three questions of the Minimal European Health Module (MEHM) which is a module that was developed to be used in EU social surveys such as the EU Statistics of Income and Living Conditions (EU-SILC) and the Eurobarometer [4]. Eurostat plays an important role in the coordination of the EU-SILC and the calculation of HLY.

A HLY improvement is the main health goal of the EU in order to reduce the social and economic burden with life expectancy lengthening [5]. The European Innovation Partnership on Active and Healthy Ageing is targeting to increase the average HLY of Europeans by two years by 2020 [3, 6]. Additionally, HLY are used as a Sustainable Development Indicator (SDI) in the Europe 2020 strategy, being the headline SDI for the public health theme [7]. The European Commission deployed efforts to harmonise the instruments and methods used in EU Member States to measure HLY and has assessed its uptake in 2006 [8]. They concluded that the use of the HLY indicator was not (yet) widespread, mainly due to limited awareness and understanding of the concept. However, the evaluation only covered the period since the adoption of HLY as a Lisbon Structural Indicator. Nonetheless, the evidence showed that the HLY indicator was seen as important to measure progress towards the Lisbon objectives, in particular because health is a precursor for economic growth and it is an instrument to put health capital higher on the European political agenda [8]. Possible methods to increase the uptake in policy and actions were identified. This included the improvement of dissemination activities by providing easier access to information and by increasing visibility.

Since 2006, no assessment has been carried out to look at the use and uptake of the GALI and HLY in the EU. Despite the EU’s attention, the evolution of the GALI and HLY indicators has not been examined for over 10 years. This study aims to assess the current use of the GALI and HLY indicators by the European Commission and EU Member States. The aim was not to cover a representative sample of potential users, but rather to have a variety of respondents active in the field who could provide a picture of current uses in Member States and the EU.

## Methods

A questionnaire was sent to experts active in the European Health and Life Expectancy Information System (EHLEIS) project, or working in a relevant public health and/or statistical area, in 28 Member states and two departments of the European Commission [9, 10]. Information was collected from a sample of 22 public health professionals, active in the field of health monitoring, belonging to agencies, such as ministries (*n* = 2), statistical offices (*n* = 8), public health institutes (n = 8) and universities (*n* = 4) that are potential users. Two experts from the European Commission responded to the survey. After purposive sampling, snowball sampling was used. The respondents were asked whether they were the right contact person to respond to the survey. If this was not the case, the respondents were asked to provide contact details for a more suitable contact person and the survey was forwarded to this person. Out of the 30 contacted persons, 23 returned their questionnaire, one suggested other colleagues to be contacted. The questionnaire was sent by email in March 2017 and a reminder was sent after one month. One response to the questionnaire per country was aimed for to provide a picture of current use. Information was complemented with data available from the EHLEIS network and the literature.

The questionnaire (Additional file 1) was developed around four topics: policy implications, use in surveys, dissemination and national capacity in staff. More specifically, the questionnaire inquired if the GALI and HLY had policy implications and was used to assess the current situation or set targets in public policy; in which survey the GALI had been introduced since 2005; how the distribution of the GALI (prevalence of participation restriction by severity level) and HLY were presented, discussed and disseminated at national or regional level; and what the national capacity was to calculate, analyse and follow-up on the GALI and HLY. The questionnaire consisted of six open-ended questions which were analysed qualitatively by using theme coding and frequency analysis. Theme coding was used for the analysis of the first and third topic in which the areas of policy and dissemination type were identified and categorised. The frequency of the use of the different types of surveys and the number of personnel working on HLY or the GALI was investigated.

## Results

Twenty-two Member States responded to the questionnaire resulting in a 79% response rate in the Member States and two responded from the European Commission. The majority of Member States respondents were part of a public health institute (*n* = 8) or a statistical institute (*n* = 8), but other agencies such as ministries and universities were also covered with respectively two and four respondents. The non-responding Member States were Croatia, Ireland, Luxembourg, Malta, Poland and Portugal. Not all answers addressed the questions completely, however only 3 out of 144 questions were left completely unanswered.

### Policy implications

Fifteen countries reported that HLY are used in the policy domain of health. In these countries, HLY are monitored and used to set targets in national health strategies or plans. In some cases, the national health plan was readjusted based on the values of the HLY. In Estonia, for example, analyses of HLY led to policy change as the mid-term assessment of the national health plan was not as good as expected and new plans were set by the Ministry of Social Affairs. In Italy, results on the indicator trends and their assessment on the impact on policies is monitored by a high-level committee and annually presented at the Italian parliament. The impact on regional policies was difficult to assess as only two respondents mentioned HLY or the GALI was used at regional level. The questionnaire revealed, HLY are usually reported in parallel with LE, however in Lithuania LE was changed to HLY as the main evaluation criteria in the program of the government. Furthermore, the GALI and HLY are used in the public policy area of disabilities and healthy ageing both by Member States and the European Commission [11, 12], but according to the respondents, more frequently in the context of sustainability and forecasting which is closely linked to budget and pension policy areas. Respondents from Belgium, Czech Republic, Estonia, Hungary, Italy, Lithuania and Latvia reported HLY to be included in the strategies of sustainable development. Also at the European Commission within the EU Sustainable Development Strategy, HLY are a headline indicator [7, 13]. In Estonia, the respondent stated that it is a government priority to increase HLY in order to increase people’s years of economic activity, which in its turn offers the possibility to increase retirement age and may ensure sustainability of the pension system. In the Netherlands, the discussion of linking pension age to HLY was raised, but eventually only LE was taken into account. In the UK, disability-free life expectancy at age 65 has been used to inform independent review into the state pension age. However, Healthy Life Expectancy (HLE), based on self-perceived health, was used to assess fairness in raising the state pension.

In the European Commission, HLY and the GALI are used in various contexts by different Directorate Generals (DGs) including DG Communication, DG Connect, DG Employment, DG Eurostat and DG SANTE (Table 1). HLY are used in the context of the European Innovation Partnership on Active and Healthy Ageing and the European Pillar of Social Rights that was adopted by the European Commission in 2017. HLY at age 65 was included as a headline indicator on the Social Scoreboard. DG Eurostat collects the GALI question in various surveys and HLY for the Sustainable Development Indicators (SDI) on public health, the quality of life indicators on health [7, 14]. The GALI is also gathered within the context of the European Disability Strategy 2010–2020 of DG Employment [15]. The GALI is used there to monitor the situation of disabled people to support the EU’s implementation of the UN Convention on the Rights of Persons with Disabilities (UNCRPD) [15, 16]. The GALI EU-SILC data is also used by the Academic Network of European Disability Experts (ANED) to produce estimations of the Europe 2020 indicators on employment and education in relation to disabled persons. HLY are also used in the Active Ageing Index by the United Nations Economic Commission for Europe and the European Commission [17]. Finally, the European Core Health Indicator on Health expectancy is also based on HLY which form the core of the quantitative indicators used in the Health at a Glance: Europe report, the Country health profiles, and DG Employment’s Joint Assessment Framework Health.

**Table 1:** Overview of the use of Healthy Life Years in the European Commission (2005–2017)

Directorate General	Use of Healthy Life Years	Measurement
DG Communication	Eurobarometer. Prevalence of activity limitation through survey instrument: GALI question.	Self-reported limitations in daily activities by age, sex and income level (SI-C11)
DG Eurostat	Metadata sheet healthy life years	Healthy Life Years at birth, at 50, at 65 and by sex
	Sustainable Development Indicator	Healthy life years and life expectancy at birth, by sex
	Quality of Life indicators on health	Healthy Life Years at birth, at 65, by sex
DG Connect and wider European Commission	European Innovation Partnership on Active and Healthy Ageing: target 2020	Healthy life years at birth, by sex
DG Employment	Social Protection & Social Inclusion	Healthy Life Years at birth, at 65, by sex (SI-C4a) Self-reported limitations in daily activities by age, sex and income level (SI-C11)
	European Pillar of Social Rights on Social Scoreboard	Healthy life years at the age of 65, by sex
	Joint Assessment Framework (JAF on health)	Healthy Life Years at birth, at 65, by sex (H-2)
	European Disability Strategy (2010–2020). Prevalence of activity limitation through survey instrument: GALI question.	Self-reported limitations in daily activities by age, sex and income level
DG Sante	Health at a Glance: Europe report, Country health profiles	European Core Health Indicators on long-term activity limitations by sex, age and educational level (ECHI 35) European Core Health Indicators on Health expectancy: Healthy Life Years at birth, at 65, by sex (ECHI 40)

### The GALI in surveys

All participants reported that the GALI question was introduced in the EU-SILC. Some respondents did point out the wording had changed over the years since its introduction. Also in the European Health Interview Survey (EHIS) most countries have the GALI question included. Only Austria reported it had removed the GALI question from the EHIS. Eight countries indicated to have the GALI question in other national or regional surveys. Only two countries mentioned to have the GALI question in the Survey of Health, Ageing and Retirement in Europe (SHARE) in their country, even though the SHARE containing the GALI question is currently carried out in 16 EU Member States. Other Surveys mentioned by participants were the censuses (*N* = 2), the European health examination survey (*N* = 2), and various other surveys on health status, health care, living style, work, school health, housing, family resources, life opportunities, gender and social protection. In Belgium, the GALI is even used in small scale studies e.g. on Lyme disease. Negotiations between Eurostat and the EU Member States are ongoing to insert the GALI as a disability variable into the Labour Force Survey once every two years, thus creating a reliable monitoring tool on the employment of people with disabilities. The 2017 SILC module on children also includes a disability perspective (the GALI variable adapted for children), thus bridging the knowledge gap regarding children with disabilities in households.

### Presentation and dissemination of HLY

Country reports are yearly produced by EHLEIS [18]. In the country reports, Member States have since 2014 the opportunity to add a one-page summary of research done within their country. This page was filled in on average by 12 of the 28 EU countries between 2014 and 2016. The country reports also contain a yearly update of a bibliography of reports and scientific papers on the GALI and HLY published within the country. Eight countries reported to have published the country reports on national websites of statistics, social affairs or of their presidency. HLY are also presented in other national reports on the state of the health of the population, well-being and pension. In France, HLY are reported in a major national plan, being the Report on New Indicators of Wealth [19]. Since HLY are measured through the GALI question in the EU-SILC or the EHIS, reports on the surveys often also mention HLY. Data on HLY are most often published on the national statistics website (*n* = 11). In the Czech Republic, a special page is even allocated to HLY on the website of the Institute of Health Information and Statistics [20]. In the UK, this is a page on comparisons and inequalities in healthy life expectancies (HLE), disability-free life expectancies (DFLE) and life expectancies (LE). The European Commission has a Eurostat metadata page allocated to HLY and reports on HLY on various other websites and in reports [14]. Also in Belgium, HLY data are presented on the website with standardised procedures for mortality analysis (SPMA) and on the website of the Belgian HIS. The latter is an interactive website where the GALI and HLY estimates are available by region [21]. Finally, several EU research groups work on HLY estimations and use it in research on health inequality, health priority setting and impact of life styles. In Lithuania for example, the university of health sciences publishes findings on HLY.

### Capacity working on the GALI or HLY

One of the main tasks of the EHLEIS project was to increase the capacity to analyse HLY within the EU through training workshops and guidelines for estimation and interpretation of HLY [8]. The EHLEIS project created a domain specific network which is currently active in all EU member states. Most countries reported to have three to four people working on the GALI and HLY. The capacity is usually distributed between the national public health institutes, the statistics offices and universities. The people involved also analyse data and calculate indicators for the EU-SILC and EHIS. HLY and the GALI are only a small part of their daily work. Three countries mentioned the capacity had increased over the years i.e. Slovenia, Czech Republic and Estonia. Four countries reported the capacity had remained unchanged i.e. Denmark, Cyprus, Greece and Lithuania. Estonia and Latvia had the highest capacity, even though the GALI and HLY are a small part among other tasks in these institutions. The Réseau Espérance de Vie en Santé (REVES) directory further informs on the research groups active in the field spread over 13 EU countries [22]. In the European Commission, there is one person involved in production of data and one person involved in methodology and dissemination, but also here the activities compose only a very small share in their total work assignments. The capacity in the European Commission is within Eurostat and is rather low at the moment. The capacity was higher during the EHLEIS project or when the Task Force on GALI was active or when specific projects related to the GALI or HLY were running. In general, the respondent from the Commission stated that there are less resources dedicated to the GALI or HLY at the moment as the production and dissemination follows already established procedures. However, some development projects are envisaged.

## Discussion

The GALI and HLY are important measures to monitor progress in various policy domains. Since the last evaluation in 2006 [8], the use of the HLY indicator has spread and gained attention and the respondents to our survey reported different uses. Analysis of the HLY have led to policy changes in e.g. Estonia and are discussed in high level political meetings in countries such as France and Italy, and at the European Commission, most recently, in the context of the European Pillar of Social Rights where HLY are a headline indicator. In Lithuania, HLY have even taken over LE as the main evaluation criteria in the program of the government. In contrast with 2006, HLY are now being used to design policies and programmes. The most widespread use of the GALI and HLY are in the area of health, but since health is acknowledged as a precursor of economic growth and important for economic decisions, HLY have gained importance in other policy areas as well. HLY and the GALI are used in at least 5 different Directorate Generals (DGs) of the Commission, including its use in the European Semester. In contrast with the report of Oortwijn et al. [8], Commission Officials do seem to be aware of the HLY indicator as its use has spread. The results from the survey show that HLY are frequently used in the context of sustainability, where HLY are reported and monitored in the strategies of sustainable development of many countries and at the European Commission. This because HLY have gained major importance in discussions on retirement age and pension. In some countries such as Estonia, it is a policy priority to increase HLY in order to increase people’s years of economic activity. The efforts of the European Innovation Partnership on Active and Healthy Aging, the Task Force on GALI and the EHLEIS project may have contributed to countries endeavours to increase HLY. Also in the policy domain of disabilities, HLY have gained political importance. Not all countries have taken up HLY systematically. In the Netherlands, for example, only LE is taken into account for pension age. Methodological issues and fluctuations are other important reasons for its limited use in e.g. Slovenia. At regional level, most recently, the Flemish region in Belgium has used healthy life years in their proposal to address social inequalities in health and wellbeing [23]. However, the policy implications at regional level seem rather limited according to the survey. This should be investigated in more depth to make any conclusive remarks as regional policy-makers were not specifically targeted in this study.

The GALI question is institutionalised in most important health questionnaires through the MEHM such as the EU-SILC, the SHARE and the EHIS [24]. This was initiated by Eurostat to have a health benchmark in all their major surveys including the non-health surveys. Interestingly, in our study, only few countries were aware the GALI was questioned through the SHARE in their country. Overall, the biggest limitation is that survey organisers use their own adaptation of the question. This leads to a variety of wording in different surveys and may limit comparability [25]. Eurostat and EHLEIS have provided support to countries with regards to harmonisation, but continuous efforts will be needed in order for the GALI question not to be altered or removed. Since the capacity has decreased in the European Commission, and the Task Force on GALI and the EHLEIS project have come to an end, it would be interesting to look at the impact of these changes on the use of the GALI and HLY in the future. In this survey, only one country reported to have removed the GALI question from the EHIS. Respondents to the survey also indicated the GALI question is used in various other surveys. Also at regional level and in smaller scale studies, the GALI has been introduced.

HLY are presented in various reports. The country reports produced by EHLEIS are well received by countries and increase the uptake of HLY at national level as the reports are published on countries websites. This is important to improve the understanding of the concept in a unified way and to provide a yearly update. Furthermore, the presentation and dissemination of HLY are most prominent on national statistics websites. Some countries provide data analysis and conclusions on their HLY results increasing the visibility of the GALI and HLY. Other findings on HLY are presented in reports on the EU-SILC and EHIS. The capacity working on the GALI and HLY in the Member States has either remained unchanged or slightly increased over the years. The capacity is usually spread over various institutions being national public health institutes, statistics offices and universities.

At the European Commission, HLY are reported in various health reports such as the Country health profiles and in Health at a glance: Europe report. Also outside the health domain, HLY are used in reports on social protection and inclusion as well as on disability and employment. The production and dissemination of HLY and the GALI follow already established procedures which confirm their institutionalisation, but has a decline in resources and capacity at EU – level as a consequence.

Since only one respondent per country was aimed for in this study, due to resources constraints, the responses may have been incomplete and may not reflect a country wide perspective. Also, the results from the study demonstrated that the areas in which the GALI and HLY are used can be broad and the respondents may only be aware of use in certain areas. However, the respondents were chosen due to their active participation in the EHLEIS network and their expertise in the field. Therefore, they seemed to be suitable to respond to the questionnaire. Participants were also asked to provide another contact person if they did not know the answers to the questionnaire. Various respondents provided links to national reports and websites. Due to the language barrier not all reports could be assessed thoroughly.

## Conclusion

In ten years, there has been a real evolution in the use of the GALI and HLY. The use of the GALI and HLY are now widespread in Member States and in the European Commission and has, over the years, become institutionalised. The GALI and HLY are used for policy making (e.g. health promotion and functional capacity of the work force), impact assessment (e.g. impact of healthy life style on health), and monitoring (e.g. trends in social protection). Additionally, HLY are increasingly being used to design policies and programmes. The GALI and HLY are not only used in the policy area of the health sector encompassing disabilities and healthy ageing, but have also gained importance in other fields such as retirement, budgets and sustainable development. The GALI is integrated in major health surveys which support its long-term sustainability. Although the capacity fluctuates between countries, most countries have capacity to evaluate the GALI and HLY. This analysis encourages further documenting for the policy makers how HLY and the GALI are measured and what type of interpretation can be driven from the secular or cross-country comparisons.

## Additional file

Additional file 1: Survey on the use of the Global Activity Limitation Indicator (GALI) and Healthy Life years (HLY) in the Member States (MS) of the European Union (EU) and associated countries. (DOCX 16 kb)
