# Supplementary material for: The use of the global activity limitation Indicator and healthy life years by member states and the European Commission
Source: Arch Public Health. 2018 Jun 28;76:30. doi: 10.1186/s13690-018-0279-z (PMC6022353; doi:10.1186/s13690-018-0279-z)
Supplement: Supplementary file 1 — Survey on the use of the Global Activity Limitation Indicator (GALI) and Healthy Life years (HLY) in the Member States (MS) of the European Union (EU) and associated countries. (DOCX 16 kb) [file 13690_2018_279_MOESM1_ESM.docx]

**Survey on the use of the Global Activity Limitation Indicator (GALI) and Healthy Life years (HLY) in the Member States (MS) of the European Union (EU) and associated countries**

This survey comprises 6 questions

**Question 1: In which areas of public policy the GALI and/or HLY are used in your country to assess the current situation and/or set targets? Can you specify if the GALY or HLY has been used to make any policy changes?**

*For instance, in many countries, the GALI and HLY are used in the domains of health and disability. In other countries, they are more widely used and concern for example the domains of employment, pensions or dependency. Finally, in some countries, they are used in more general economic and social policies such as in France where they are included in the 10 new indicators of wealth or in Belgium where they are included in the 64 complementary indicators to GDP.*

*In France, the Law of 13 April 2015 states that the Government shall submit annually to Parliament a report presenting new indicators of wealth, such as indicators of inequality, quality of life and sustainable development, over the past few years. Disability-Free Life Expectancy (HLY) is one of the 10 new indicators of wealth that make up this report.*

*In Belgium, the Law of 14 March 2014 instructs the National Accounts Institute to develop a set of indicators to measure quality of life, human development, social progress and the sustainability of the economy. Healthy Life Expectancy (HLY) is one of 64 indicators complementary to GDP examined annually by the Federal Planning Bureau*

**In which areas of public policy GALI and/or HLY are used in your country to assess the current situation or set targets? Can you specify if the GALY or HLY has been used to make any policy changes?**

Please list the areas of public policies and, for each area, specify the level (national, regional, etc.), name the departments or agencies in charge of these policies, indicate set targets and references (reports, website, etc.) and, if possible, provide a contact

ADD AS MANY LINES AS YOU NEED

If you are not the right person to answer this question, please provide the right contact

**Question 2: In which surveys the GALI has been introduced in your country since 2004/2005?**

*In several countries, the GALI is not only used in EU-SILC and in SHARE but also in various national and sub-national surveys. Some countries may have introduced the GALI in their census.*

**In which surveys the GALI has been introduced in your country since 2004/2005?**

For each survey, please, name the survey and the agency in charge of it, specify the level (national, regional, etc.), indicate references (reports, website, etc.), provide, if possible, the wording of the GALI and a contact for the survey/census

ADD AS MANY LINES AS YOU NEED

If you are not the right person to answer this question, please provide the right contact

**Question 3: Is the prevalence of activity limitations and HLY regularly presented and discussed in national and/or regional health and disability reports in your country?**

*Several countries regularly report on the prevalence of activity limitations, based on the GALI instrument, as well as on the HLY in health and disability reports.*

**Is the prevalence of activity limitations and HLY regularly presented and discussed in national and/or regional health and disability reports in your country?**

For each report or series of reports, please indicate its title, the agency in charge of it and its references, including associated website, especially if the report is available on line

ADD AS MANY LINES AS YOU NEED

If you are not the right person to answer this question, please provide the right contact

**Question 4: Did you produced in your country materials (4-pages, policy-briefs, etc.) introducing the GALI and HLY to a wider audience (policy makers, media, politicians, teachers, etc.)?**

*Some countries produced 4-pages and/or policy-briefs to introduce the GALI and HLY to the media and/or the politicians.*

**Did you produced in your country materials (4-pages, policy-briefs, etc.) introducing the GALI and HLY to a wider audience (policy makers, media, politicians, teachers, etc.)?**

If yes, can you list this material with references (associated web-site, contacts, etc.)? Can you provide, if possible, a copy of this material?

ADD AS MANY LINES AS YOU NEED

If you are not the right person to answer this question, please provide the right contact

**Question 5: Are information and results about GALI and HLY available on websites in your country?**

*In several countries information and results about GALI and HLY are available on websites.*

**Are information and results about GALI and HLY available on websites in your country?**

If yes, can you list the websites with their address? Can you indicate the department / agency in charge of it and, if possible, provide a contact?

ADD AS MANY LINES AS YOU NEED

If you are not the right person to answer this question, please provide the right contact

**Question 6: Has the capacity to calculate, to analyze and follow-up on the GALY and/or HLY changed over the years in your country? Can you indicate how many people are involved/work on the GALI/HLY in your country?**

If yes, can you list the websites with their address? Can you indicate the department / agency in charge of it and, if possible, provide a contact?

ADD AS MANY LINES AS YOU NEED

If you are not the right person to answer this question, please provide the right contact

**If you are not the right contact to answer this survey**

Please list better contacts, indicating their names, institutions and email address

**THANKS A LOT FOR YOUR PARTICIPATION**
